# ACTIN7 Is Required for Perinuclear Clustering of Chloroplasts during Arabidopsis Protoplast Culture

**DOI:** 10.3390/plants9020225

**Published:** 2020-02-10

**Authors:** Michael B. Sheahan, David A. Collings, Ray J. Rose, David W. McCurdy

**Affiliations:** School of Environmental and Life Sciences, The University of Newcastle, Callaghan, NSW 2308, Australia; Michael.Sheahan@newcastle.edu.au (M.B.S.); David.Collings@newcastle.edu.au (D.A.C.); Ray.Rose@newcastle.edu.au (R.J.R.)

**Keywords:** actin, *ACTIN* genes, Arabidopsis, chloroplast clustering, protoplast culture

## Abstract

In Arabidopsis, the actin gene family comprises eight expressed and two non-expressed *ACTIN* (*ACT*) genes. Of the eight expressed isoforms, *ACT2*, *ACT7,* and *ACT8* are differentially expressed in vegetative tissues and may perform specific roles in development. Using tobacco mesophyll protoplasts, we previously demonstrated that actin-dependent clustering of chloroplasts around the nucleus prior to cell division ensures unbiased chloroplast inheritance. Here, we report that actin-dependent chloroplast clustering in Arabidopsis mesophyll protoplasts is defective in *act7* mutants, but not *act2-1* or *act8-2*. *ACT7* expression was upregulated during protoplast culture whereas *ACT2* and *ACT8* expression did not substantially change. In *act2-1*, *ACT7* expression increased in response to loss of *ACT2*, whereas in *act7-1*, neither *ACT2* nor *ACT8* expression changed appreciably in response to the absence of ACT7. Semi-quantitative immunoblotting revealed increased actin concentrations during culture, although total actin in *act7-1* was only two-thirds that of wild-type or *act2-1* after 96 h culture. Over-expression of *ACT2* and *ACT8* under control of *ACT7* regulatory sequences restored normal levels of chloroplast clustering. These results are consistent with a requirement for ACT7 in actin-dependent chloroplast clustering due to reduced levels of actin protein and gene induction in *act7* mutants, rather than strong functional specialization of the ACT7 isoform.

## 1. Introduction

The actin cytoskeleton facilitates numerous cellular processes required for the correct functioning and development of multicellular eukaryotes. Unlike yeast, where actin is encoded by a single gene [1], in multicellular eukaryotes, actins are encoded by multi-gene families. In *Arabidopsis thaliana* (Arabidopsis), eight expressed actin isoforms exist [2]. Based on their phylogenetic relationship and expression pattern, Arabidopsis actins are classified as either vegetative or reproductive, with each class being expressed predominantly in vegetative or reproductive tissues, respectively. The vegetative class of actins comprises ACT2, ACT7, and ACT8. Interestingly, the sequence divergence between individual actin isoforms in plants is greater than the divergence between non-muscle and muscle isoforms of actin in animals, suggesting the potential for plant actin isoforms to perform discrete functions within the cell [3,4]. Alternately, such divergence may simply reflect developmental rather than intracellular specialization. While the number of actin genes varies dramatically between different plant species, with the eudicot *Medicago truncatula* containing only four actin genes but with the monocot *Zea mays* containing 21 separate genes, the division of actins into vegetative and reproductive classes is broadly conserved across plant species [4,5]. 

In animal and fungal cells, different actin isoforms can perform different functions, not only between the major classes of muscle and non-muscle actin but even within these classes, with these conclusions derived from a range of molecular and biochemical experiments [6], green fluorescent protein (GFP)-fusion studies [7] and mutant analyses [8]. Evidence for isoform-specific functions of plant actins is strongest for the vegetative actin ACT7. During tissue culture, the *ACT7* gene is strongly induced by auxin and is required for callus formation, whereas formation of callus proceeds normally in an *ACT2* knockout mutant [9]. *ACT7* mutants also show delayed germination and altered root growth, possibly explaining the strong selective disadvantage of *act7-4* when grown in competition with wild-type plants [10]. Nuclear migration and positioning to establish polar outgrowth of root hairs requires ACT7 [11], whereas ACT2 is required for bulge site selection and tip growth [12]. Furthermore, an *ACT2*-dependent defect in root hair growth could not be complemented by overexpression of *ACT7* in the *act2* mutant [13]. Bacterially-expressed ACT2 and ACT7 display distinct biochemical properties such as kinetics of filament polymerization and interaction with actin-binding proteins such as profilin [14]. Recently, experiments using novel GFP-actin fusions demonstrated that ACT2 and ACT7 generate distinct and cell-type-specific filamentous arrays, either forming isoform-specific filaments or various mixed-polymer filaments depending on the cell type [15]. Collectively, these studies point towards functional specificity of the actin cytoskeleton in plants by expressing functionally different actin isoforms [3]. 

Progress in investigating potential isoform-specific functions of plant actins has been limited by the paucity of point mutations in different *ACT* genes. Unlike the situation with tubulin and microtubules, where extensive collections of tubulin mutants with either recessive or semi-dominant growth phenotypes exist in both Arabidopsis [16,17] and other species including rice [18] and tef [19], few actin mutants have been described. However, in recent years several actin mutants have been characterized in Arabidopsis and other species. These include the dominant negative *act2-2D* mutant in *ACT2* which showed disruption not just in root hair growth but also in diffusely elongating cells, the recessive *der1* mutants in *ACT2* in which different point mutations all result in defects in root hair growth [12] and changes in overall growth of the plant [20], and the dominant-negative *fiz1* point mutation in *ACT8* which results in fragmentation of the actin cytoskeleton and disrupted organelle trafficking [21].

During culture of tobacco mesophyll protoplasts, chloroplasts move from the cortical cytoplasm and reposition to the perinuclear region [22]. This process of chloroplast clustering to the nucleus ensures that when the protoplasts subsequently divide, unbiased inheritance of the chloroplast population to each daughter cell is achieved [22,23]. Experiments with inhibitors of either microtubule or actin filament (AF) polymerization indicated that this repositioning is an AF- and not microtubule-dependent phenomenon [22]. Time-lapse imaging suggested that chloroplast movements from the peripheral cytoplasm to the perinuclear region may involve the capture of individual chloroplasts in an actin network, which itself undergoes dynamic repositioning [23]. This process of bulk repositioning of chloroplasts enmeshed in a dynamic network of actin is distinct from the more conventional models whereby dynamic chloroplasts move along stationary AFs or bundles [24]. Once repositioned to the perinuclear region, however, the mechanism that results in the chloroplasts being maintained there remains unknown. A likely possibility is that the chloroplasts are trapped somehow by specific interaction with the “nuclear basket” of AFs, which is a common component of the actin cytoskeleton in plant cells [3,22,24].

In this study, we have investigated the process of chloroplast clustering in the model species Arabidopsis. We show that as with tobacco, chloroplasts in cultured Arabidopsis mesophyll protoplasts undergo chloroplast clustering to the perinuclear region prior to cell division, and that this process is dependent on the actin cytoskeleton but not microtubules. Chloroplast clustering was completely absent in two mutant alleles of *ACT7*, *act7-1,* and *act7-4*, but was essentially unaffected in mutants of the other vegetative actins. While reverse transcription-quantitative polymerase chain reaction (RT-qPCR) and immunoblotting demonstrated that both *ACT7* expression and total actin accumulated during protoplast culture, this did not happen in the *act7* mutants and total actin levels remained low. Further, as over-expression of *ACT2* and *ACT8* under control of the *ACT7* promoter restored full chloroplast clustering, this indicates that the loss of chloroplast clustering in the *act7* mutants is probably due to reduced levels of total actin. These results are discussed in terms of the mechanism of actin-dependent chloroplast clustering and the failure of this process in the *act7* mutants.

## 2. Results

### 2.1. Actin-Dependent Clustering of Chloroplasts during Protoplast Culture

**Figure 1:**
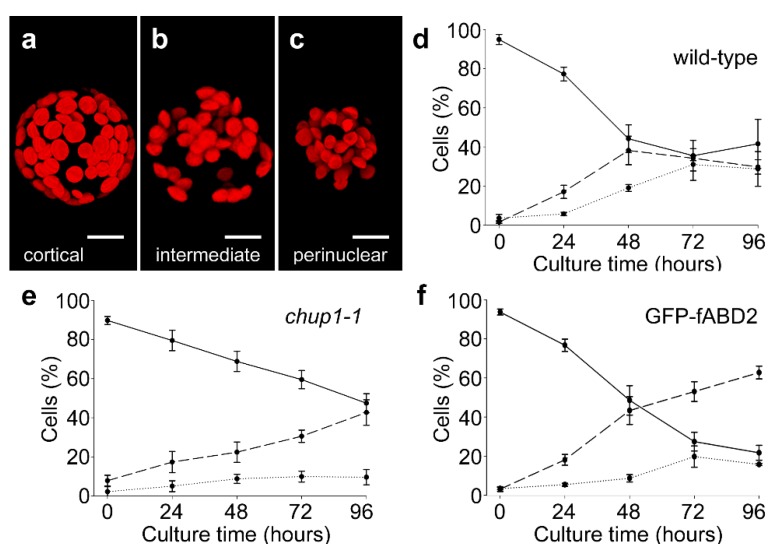
Analysis of chloroplast clustering in Arabidopsis protoplasts. (**a**–**c**) Images of chloroplasts (red autofluorescence) in either the (**a**) cortical, (**b**) intermediate, or (**c**) perinuclear distributions in cultured protoplasts. (d-f) Quantitative analysis of the repositioning of chloroplasts from cortical to perinuclear across 96 h of culture. (**d**) Wild-type protoplasts. (**e**) *chup1-1* protoplasts. (**f**) Protoplasts from a GFP-fABD2 line. For d–f, solid lines, dashed lines and dotted lines represent cortical, intermediate and perinuclear distributions of chloroplasts, respectively. Scale bars in a–c = 10 µm. Data in d-f is mean ± SE, *n* = 3–5 experiments.

Prior to the first cell division in cultured tobacco mesophyll protoplasts, chloroplasts move via an actin-dependent process from the cortical cytoplasm and cluster around the surface of the centrally-placed nucleus [22]. We confirmed that a similar relocation and perinuclear clustering of chloroplasts occurred in cultured Arabidopsis mesophyll protoplasts (Figure 1). Immediately following protoplasting, chloroplasts were present primarily in the cortex of protoplasts (Figure 1a) but over the course of four days they relocated through intermediate localizations (Figure 1b) to a primarily perinuclear location (Figure 1c). These stages of re-localization were quantified in wild-type protoplasts (Figure 1d) and this pattern was not affected by the expression of the fluorescent F-actin marker GFP-fABD2 (a fusion of GFP with actin binding domain 2 of Arabidopsis AtFIM1 [25]) (Figure 1f).

To examine whether the mechanism of chloroplast clustering in cultured protoplasts is similar to light-dependent chloroplast repositioning, we analyzed clustering in mesophyll protoplasts isolated from homozygous mutants of *CHUP1*. CHUP1 is required for light-dependent repositioning of chloroplasts from anticlinal to periclinal cell walls of leaf mesophyll cells exposed to different intensities of blue light [26]. In the *chup1-1* mutant, perinuclear clustering of chloroplasts continued to occur in cultured mesophyll protoplasts (Figure 1e), with this being only slightly slower than wild-type (Figure 1d). This result indicates that the mechanisms for repositioning chloroplasts in mesophyll cells in response to the stimuli of high light and protoplast culture are substantially different.

**Figure 2:**
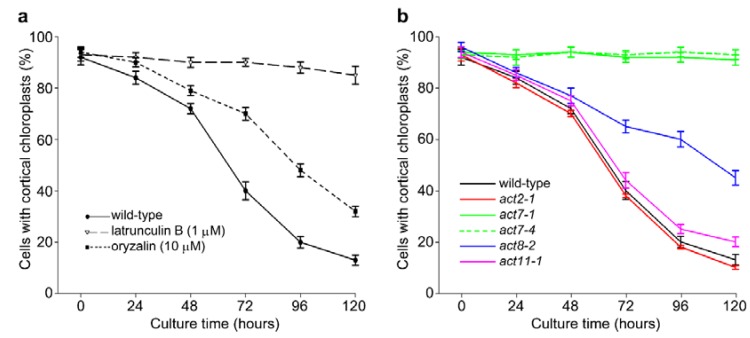
Chloroplast clustering in Arabidopsis protoplasts is actin-dependent and disrupted in *act7* mutants. (**a**) Clustering of chloroplasts occurs in Arabidopsis protoplasts. Latrunculin B (1 µM) inhibited clustering, showing actin-dependence, whereas oryzalin (10 µM) showed that clustering is independent of microtubules. (**b**) Clustering of chloroplasts around the nucleus is unaffected in *act2-1* or *act11-1* protoplasts, is completely inhibited in *act7* alleles, *act7-1* and *act7-4*, and is slowed in *act-8-2* protoplasts. Data is mean ± SE, *n* = 3–5 experiments.

As found in tobacco, culturing Arabidopsis protoplasts in the presence of the actin disrupter latrunculin B (1 µM) prevented relocation of chloroplasts from the cortex, demonstrating that relocation is dependent on the actin cytoskeleton. In contrast, microtubule de-polymerization with 10 µM oryzalin slowed but did not block the clustering response (Figure 2a). To test the involvement of different actin isoforms in mediating chloroplast clustering, we performed similar analyses using T-DNA insertional mutants of all three Arabidopsis vegetative actins (*ACT2*, *ACT7,* and *ACT8*) and one reproductive actin (*ACT11*). ACT2 and ACT7 are the most divergent vegetative actins, while ACT8 is closely similar to ACT2. Each insertion line shows substantial or complete reduction in the levels of the respective *ACT* transcript and protein product in seedling tissue [9,10,13,27]. When mesophyll protoplasts from each line were cultured for up to five days, chloroplast clustering in *act2-1* and *act11-1* protoplasts occurred to the same extent as in wild-type protoplasts (Figure 2b), whereas no clustering was evident in protoplasts from the *ACT7* alleles, *act7-1* and *act7-4* (Figure 2b). Clustering did occur in *act8-2* protoplasts, but at a slower rate than the wild-type. Overall, these results suggest that the phenomenon of chloroplast clustering relies on the presence of functional ACT7 protein.

**Figure 3:**
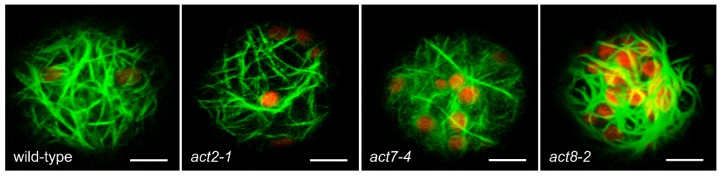
Actin networks in wild-type and *act* mutants. Images are maximum projections of optical sections (4 µm deep) of 48-h protoplasts derived from wild-type (Col-0) and *act* mutants expressing GFP-fABD2. Red is chlorophyll autofluorescence from chloroplasts. Scale bars = 10 µm.

To determine whether the architecture of the actin cytoskeleton was compromised in the actin mutants, we crossed *act2-1*, *act7-4,* and *act8-2* plants with a line expressing GFP-fABD2 [25]. Analysis of protoplasts cultured for 48 h showed that actin organization was largely intact in *act2-1* and *act7-4* compared to wild-type, but that *act8-2* revealed larger bundles of cortical actin. In all lines except *act8-*2, a finer cortical actin network was apparent along with larger subcortical bundles of AFs (Figure 3).

### 2.2. ACT7 Is Up-Regulated before Re-Initiation of Cell Division

**Figure 4:**
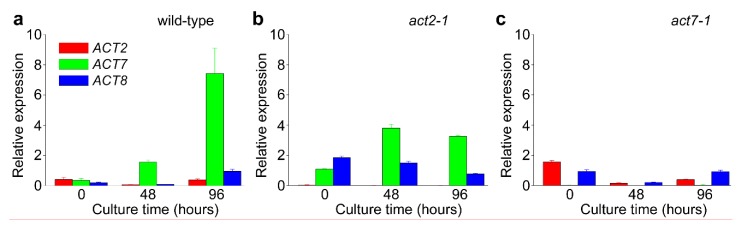
Changes in relative expression of *ACT2*, *ACT7,* and *ACT8* across culture of protoplasts derived from (**a**) wild-type and (**b**) *act2-1* and (**c**) *act7-1* mutants. Data is mean ± SE, *n* = 3 experiments.

To assess temporal changes in *ACT* gene expression across protoplast culture, real-time qPCR was performed (Figure 4). Of the three vegetative actin genes, *ACT7* expression in wild-type (Col-0) was substantially increased (over six-fold) over 96 h of protoplast culture, whereas *ACT2* and *ACT8* levels did not change appreciably. In *act7-1* mutant protoplasts, *ACT2* and *ACT8* expression did not change in response to the loss of *ACT7*, whereas in the *act2-1* mutant, expression of *ACT7* and to a lesser extent *ACT8* increased to some degree in response to the lack of *ACT2* (Figure 4). These results show that *ACT7* transcription increases as cells prepare to re-enter the cell cycle and suggests it is change in *ACT7* expression that contributes to the perinuclear clustering of chloroplasts in cultured protoplasts.

### 2.3. Analysis of Total Actin

**Figure 5:**
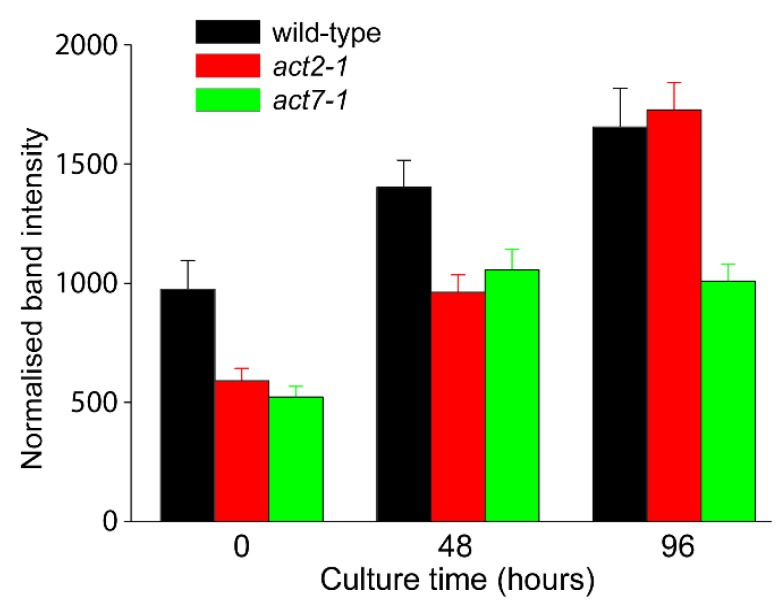
Semi-quantitative immunoblotting of total actin levels across culture of protoplasts derived from wild-type (Col-0), *act2-1,* and *act7-1*. Total protein was extracted from cultured protoplasts at the indicated time points and immunoblotting was performed with C4 anti-actin monoclonal antibody. Normalised band intensities were determined by duplicate immunoblots probed with anti-ß-tubulin. Data is mean ± SE, *n* = 3 experiments.

Given the changes in gene expression documented in Figure 4, semi-quantitative immunoblotting was performed to compare total levels of actin in wild-type compared to *act* mutants. As isoform-specific anti-actins that distinguish between ACT7, ACT2, and ACT8 polypeptides are not available, see [24], total actin in protoplasts was assessed using the C4 monoclonal anti-chicken gizzard actin antibody [28]. In freshly isolated protoplasts, total actin levels in the *act2-1* and *act7-1* mutants were substantially decreased compared to wild-type (Figure 5). Across subsequent protoplast culture, total actin levels in wild-type protoplasts increased in a linear fashion, whereas total actin levels in the *act2-1* mutant was about two-thirds that of wild-type at 48 h but equaled wild-type levels by 96 h culture. In contrast, increase in total actin levels in *act7-1* plateaued by 48 h and remained at about two-thirds that of both wild-type and *act2-1* by 96 h (Figure 5). This result shows that total actin levels in the *act7-1* mutant did not respond to protoplast culture in the same way as wild-type and *act2-1*, suggesting that the chloroplast clustering phenotype seen in the *act7* mutants is most likely due to decreased total protein levels in the mutant rather than resulting from an isoform-specific function of *ACT7.* Interestingly, while total actin levels in both *act2-1* and *act7-1* were similarly decreased compared to wild-type at 48 h, the reduction in total actin levels in *act2-1* had no effect on chloroplast clustering at 48 h whereas a similar reduction in total *act7-1* was associated with an inhibition of chloroplast clustering (Figure 2).

### 2.4. Over-Expression of Vegetative Actins Suppresses the Act7-4 Phenotype 

**Figure 6:**
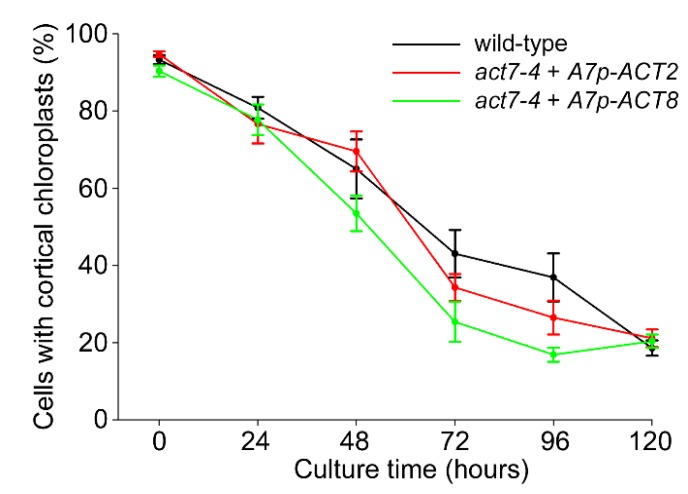
Over-expression of the vegetative actins *ACT2* and *ACT8* suppresses the *act7-4* phenotype. Chloroplast clustering is indistinguishable between wild-type or two transgenic lines whereby *ACT2* and *ACT8* are expressed in the *act7-4* mutant under control of the *ACT7* promoter. Data is mean ± SE, *n* = 3 experiments.

Given the suggestion from gene expression and total protein data that reduced actin levels within the *act7* mutants may cause the chloroplast clustering phenotype, we tested whether over-expression of the two vegetative actins *ACT2* and *ACT8*, under the control of the *ACT7* promoter and terminator sequences [24], can complement the *act7* phenotype. Analysis of different lines in which *ACT7p::ACT2* and *ACT7p::ACT8* constructs were expressed in the *act7-4* mutant showed that over-expression of either *ACT2* or *ACT8* restored normal chloroplast clustering in the *act7-4* mutant compared to wild-type (Figure 6). This result is consistent with the interpretation that it is the actin protein concentration itself, rather than a functional dependence-specific actin isoform, that causes the disrupted chloroplast clustering phenotype in the *act7-1* and *act7-4* mutants.

## 3. Discussion

The perinuclear clustering of chloroplasts which occurs prior to cell division in protoplasts provides a mechanism to ensure unbiased inheritance of the chloroplast population to both daughter cells. This process was first reported in tobacco mesophyll protoplasts [22] and this report now establishes that the same phenomenon occurs in mesophyll protoplasts from Arabidopsis leaves. Furthermore, as found in tobacco, the actin inhibitor latrunculin B strongly prevented chloroplast clustering from occurring, indicating that the process in Arabidopsis protoplasts is also actin-dependent. 

The mechanism of actin-dependent chloroplast repositioning during protoplast culture is not known. Time-lapse movies of tobacco protoplasts expressing GFP-fABD2-labelled AFs showed chloroplasts enmeshed within an actin network, with localized movement of the actin network itself appearing to be the driving force for re-location of the chloroplasts, rather than movement of chloroplasts along stationary actin bundles. Furthermore, chloroplasts within the perinuclear region appeared to be enmeshed within a static perinuclear actin network [23]. These observations are distinct from descriptions of other examples of chloroplast movement in which chloroplasts enmeshed in actin baskets nonetheless translocate along stationary AFs [24]. Light-dependent re-location of actin bundles is reported to involve short, chloroplast-associated filaments, described as chloroplast actin, which assemble at the leading edge of chloroplasts [29] and interact with the plasma membrane to relocate chloroplasts in response to light, reviewed in [30]. Interestingly, the presence of this chloroplast actin and its interaction with the plasma membrane is dependent on CHUP1, a multifunctional protein required for proper chloroplast positioning and photo-relocation movements [26]. Since the process of chloroplast clustering observed here was not substantially disrupted in the *chup1* mutant, the mechanism involved in chloroplast clustering must be different from light-dependent chloroplast relocation.

We tested the involvement of individual actin isoforms in the process of chloroplast clustering in Arabidopsis by analyzing various *ACT* knockout mutants. Being insertional mutants into the promoters, these mutants have a considerable or complete reduction in the levels of their respective *ACT* transcripts and protein products [9,10,13,27]. Somewhat surprisingly, clustering was entirely absent in two alleles of *act7* (*act7-1* and *act7-4*) but was unperturbed in *act2-1* and *act11-1* and only slowed in *act8-2*. The organization of the actin network was not substantially different to that of wild-type in transgenic lines expressing the GFP-fABD2 marker for actin filaments (Figure 3). 

*ACT7* is a functionally diverse vegetative actin. Unlike *ACT2,* which is constitutively expressed in the vegetative tissues of adult plants, *ACT7* is expressed more strongly in younger tissues, and is responsive to external stimuli and hormones [3,4,9,13]. This ability for ACT7 to show highly regulated expression is consistent with the strong upregulation of its expression during protoplast culture. A consequence of this variable expression is also that the various *act7* mutants are defective in aspects of root growth and development [10,11]. Our quantitative gene expression analysis showed that *ACT7* was the dominant *ACT* gene expressed across culture of wild-type protoplasts (Figure 4) and that the expression of *ACT7* along with *ACT8* partially compensated for the absence of *ACT2* in the *act2-1* mutant. In contrast, neither *ACT2* nor *ACT8* expression changed substantially from wild-type to compensate for the loss of *ACT7* (Figure 4). This observation suggests that the absence of chloroplast clustering in the *act7* mutants was due to the absence of any compensatory expression of ACT isoforms in *act7*. This conclusion is supported by the semi-quantitative immunoblotting (Figure 5), which demonstrated that total actin abundance increased across protoplast culture in wild-type but that actin levels in *act7-1* were about two thirds that of wild-type. Thus, the absence of compensatory *ACT* expression resulting in reduced total actin levels in *act7-1* may be the explanation for loss of chloroplast clustering in this mutant. 

Our conclusion that it is a reduction in overall actin concentration, rather than the functional specialization of actin isoforms, that is responsible for the inhibition of chloroplast clustering in *act7* mutants is also supported by the over-expression experiments in which the two vegetative actins, ACT2 and ACT8, were expressed in the *act7-4* mutant under control of *ACT7* regulatory sequences. In both cases, chloroplast clustering recovered to wild-type levels across 120 h of protoplast culture (Figure 6). This result, along with the total actin levels reported by immunoblotting, clearly establishes that the absence of chloroplast clustering in *act7* mutants is due to reduced levels of total actin in *act7* protoplasts rather than being caused by isoform-specific, functional specialization of ACT7. A similar conclusion was reached following experiments in which the sensitivity of root elongation and root swelling to low doses of latrunculin was tested in wild-type and *act* mutant lines. Wild-type plants showed reductions in root elongation and root tip swelling following 48 h treatments with latrunculin B concentrations of 100 nM and higher (data not shown), equivalent to a previous report [31]. In *act7-1* and *act2-1*, however, induced swelling and reduced elongation responses were observed at concentrations as low as 30 nM. Importantly, preliminary experiments have shown that in *ACT7p::ACT2* plants in which *ACT2* was expressed under control of the *ACT7* promoter, the mutant phenotype was complemented with plants showing wild-type responses to latrunculin treatments (data not shown). Thus, the disrupted chloroplast clustering phenotype in the *act7* mutants is not the only phenotype that can be rescued, suggesting a common mechanism based on the total levels of ACT protein. 

This ability to complement *act* mutant phenotypes by over-expression of other *ACT* genes, suggesting functional interchangeability of ACT proteins, has previously been demonstrated in Arabidopsis. For example, the *act7-4 act8-2* double mutant shows a strong and diverse growth phenotype: over-expression, however, of protist and animal cytoplasmic *ACT* genes under control of the ACT2 promoter complemented the mutant phenotype showing that these divergent actins retained functional competence, although animal muscle actin did not have this ability [3]. These results all suggest that the vegetative actin genes, *ACT2*, *ACT7,* and *ACT8*, which are expressed in Arabidopsis, need not have any functional specializations. Instead, their role might be to show different patterns of expression within the plant and differences in responsiveness to external stimuli. A counter view to this conclusion, however, is that functional differences do exist between vegetative actin isoforms. This has been suggested based on observations of biochemical differences between ACT2 and ACT7 [14], and the structurally different arrays and filaments formed by these two vegetative actins [15]. Moreover, while our data broadly support the concept that it is total actin levels rather than isoform functional specialization that are responsible for the *act7* mutant phenotype, the lack of a phenotype in the *act2-1* mutant even when total ACT levels are low may suggest some subtle functional differences between the different isoforms. 

The observation that total actin levels are responsible for the *act7* clustering phenotype presumably also has implications for the mechanism of chloroplast clustering. As stated previously, the collective movement of chloroplasts enmeshed within a dynamic actin network [23] is different from other models of actin-dependent motility in plant cells [24,30]. In this regard, the reduction in total actin levels seen in the *act7-4* mutant may be a critical feature that does not support differential enmeshment of chloroplasts undergoing clustering, and the more static network of perinuclear actin which appears to be responsible for trapping motile chloroplasts at the nuclear surface. Presumably, diverse activities of actin-binding proteins interacting with sub-domains of the actin cytoskeleton in cultured protoplasts may be the mechanistic element responsible for this process, however minimal levels of total actin in each protoplast is required to maintain differential organization of the actin cytoskeleton and thus execute the ability to differentially orchestrate organelle movements as seen in the example of chloroplast clustering.

## 4. Materials and Methods

### 4.1. Plant Growth, Protoplast Isolation and Culture and Reagents

*Arabidopsis thaliana* (Arabidopsis) seeds (Col-0) were surface sterilized in 70% *(v/v*) ethanol for 1 min followed by a 1:4 dilution of White King^TM^ commercial bleach for 5 min, then extensive washed in sterile water and suspended in 0.1% (*w/v*) agar solution. The seeds were then distributed evenly over the surface of Petri dishes containing half-strength Murashige and Skoog medium supplemented with 1% (*w/v*) sucrose and solidified with 1.2% (*w/v*) Bacto agar. Plates were placed at 4 °C for 2 days before being placed horizontally in a controlled growth environment of 16/8 h day/night (22 °C/18 °C) and fluence rate of 90–120 μmol m^2^ s^1^. After 10–14 day growth, the plates were placed in complete darkness for 24 h to reduce starch levels in chloroplasts. The aerial portion of plants was then excised with sterile scissors, placed on sterile paper towel, and coarsely macerated with a sterile razor blade. Protoplasts were then isolated from the macerated leaf tissue and cultured for up to 120 h as described previously for tobacco mesophyll protoplasts [22]. All reagents were purchased from Sigma-Aldrich (Sydney, NSW, Australia) unless specified otherwise. Oryzalin (Crescent Chemical, Singapore) and latrunculin were prepared as 1000× stocks in dimethylsulfoxide (DMSO), and diluted to 10 µM and 1 µM, respectively. Protoplast cultures were exposed throughout the culture period to the various drugs, using 0.1% (*v/v*) DMSO as control.

Different *act* insertional mutant lines (obtained from Prof. Richard Meagher, University of Georgia) were crossed with a line expressing *GFP:fABD2* [25]. The *chup1-1* mutant was generously supplied by Prof. Masamitsu Wada (Kyushu University).

### 4.2. Microscopy 

Protoplasts were placed in welled slides in a 2:1 ratio with 0.5% (*w/v*) agarose (agarose type VII, Sigma) before applying a coverslip. Images of protoplasts were acquired as z-series with a 1 µm interval using a Zeiss LSM510 confocal laser-scanning microscope equipped with a 40× water-immersion objective. GFP was viewed with 488 nm excitation and barrier filter selecting 500 to 530 nm, while chloroplast autofluorescence was viewed with 543-nm excitation and a long-pass filter selecting above 650 nm. 

### 4.3. Reverse Transcription-Quantitative PCR (RT-qPCR)

Protoplasts of the various lines under investigation were cultured for the indicated times, then 8 × 10^4^ protoplasts were pelleted and dissolved in RLT buffer for total RNA isolation using the Qiagen Plant RNeasy kit (Qiagen, Hilden, Germany). Synthesis of cDNA from 1 μg of total RNA was performed using a Superscript III kit (Invitrogen, Waltham, MA, USA) following the manufacturer’s instructions. Gene expression was analyzed by RT-qPCR using a RotorGene-Q (Qiagen). PCR reactions were carried out using *UBC21* (At5G25760) as the reference gene. PCR master mixes were prepared with Platinum Taq (Invitrogen) using the provided buffer supplemented with 1.5 μM SYTO9 (Invitrogen), 3 mM dNTPs and 0.4 μM of each primer. The qRT-PCR cycling conditions comprised an initial denaturation at 95 °C for 2 min followed by 40 cycles of 95 °C for 10 s, 60 °C for 30 s and 72 °C for 30 s. For each gene analyzed, two biological and three technical repetitions were performed. Data analyses were performed using Q-Gene software [32,33,34]. Q-Gene software uses mean normalized data and the ΔΔ*C*_T_ method to calculate relative expression (the calibrator was the lowest expression point for the gene investigated) and standard errors. Primers used for RT-qPCR are listed in Table S1 and amplification efficiency based on serial dilution was greater than 90% for each primer pair (Table S1). Table S2 shows the expression data of *UBC21* (At5G25760) used as the reference gene at 0, 48 and 96 h protoplast culture.

### 4.4. Immunoblotting

For immunoblotting experiments, Col-0, *act2-1,* and *act7-1* protoplasts were cultured for the indicated times and 1 × 10^6^ protoplasts pelleted and dissolved in SDS sample buffer and heated to 95 °C for 5 min. After centrifugation at 10,000× *g* for 5 min, equal volumes of supernatant from each extract were loaded into wells of 10% polyacrylamide gels. After electrophoresis and transfer to nitrocellulose, the blots were blocked with 5% (*w/v*) skim milk for 1 h, washed in Tris-buffered saline solution then probed with C4 anti-chicken gizzard actin (MP Biomedicals, Irvine, CA, USA; [27]) for 2 h. After washing with Tris-buffered saline (TBS), the nitrocellulose was incubated with goat anti-mouse secondary antibody coupled to alkaline phosphatase. Color development was by Western Blue (Promega, Madison, WI, USA) and individual band intensities were quantified from scanned images using ImageJ. Normalized band intensities were determined by comparison to total tubulin levels (mouse monoclonal anti-α-tubulin, clone B512, Sigma) assessed on duplicate nitrocellulose blots and processed in parallel. 

## Supplementary Materials

The following are available online at https://www.mdpi.com/2223-7747/9/2/225/s1, Table S1: List of primers used in this study. Table S2: Expression of *UBC21* in isolated protoplasts cultured for 0, 48 and 96 h.
