# Supplementary material for: ACTIN7 Is Required for Perinuclear Clustering of Chloroplasts during Arabidopsis Protoplast Culture
Source: Plants (Basel). 2020 Feb 10;9(2):225. doi: 10.3390/plants9020225 (PMC7076399; doi:10.3390/plants9020225)
Supplement: Supplementary file 1 [file plants-09-00225-s001.pdf]

Table S1. PCR primers used in this study.

Gene name	AT number	Direction	Sequence	Tm (°C)	Amplicon size (bp)	Efficiency (%)
UBC21	At5G25760	forward	CTGCGACTCAGGGAATCTTCTAA	64.8	60	92.5
		reverse	TTGTGCCATTGAATTGAACCC	62.8		
ACT2	At3G18780	forward	TTCCAGCAGATGTGGATCTC	62.4	313	91.0
		reverse	TGTCACACACAAGTGCATCA	63.2		
ACT7	At5G09810	forward	TCCAACAGATGTGGATTTCAA	60.9	114	92.1
		reverse	AACAAACTCACCACCACGAA	63.2		
ACT8	At1G49240	forward	TTCCAGCAGATGTGGATCTC	62.4	232	93.0
		reverse	TTTCAAACCTGCTCCTCCTT	62.5		

Table S2. Ct values of *UBC21* used in RT-qPCR analysis of actin gene expression in this study. Data shows mean Ct ± SE of *UBC21*expression in wild type, *act2-1* and *act7-1.*

0 h	48 h	96 h
26.4 ± 0.7	28.0 ± 0.3	27.8 ± 0.2
